# Endocrine therapy in prostate cancer: time for reappraisal of risks, benefits and cost-effectiveness?

**DOI:** 10.1038/bjc.2012.523

**Published:** 2012-11-29

**Authors:** L Bourke, P Kirkbride, R Hooper, A J Rosario, T J A Chico, D J Rosario

**Affiliations:** 1 Department of Primary Care and Public Health, Barts and The London School of Medicine and Dentistry, Queen Mary University of London, LondonE1 2AT, UK; 2 Consultant Clinical Oncologist, Weston Park Hospital, Sheffield S10 2SJ, UK; 3 General Practitioner, Foxhill Medical Centre, Sheffield S6 1AF, UK; 4 NIHR Cardiovascular Biomedical Research Unit, Sheffield Teaching Hospitals NHS Foundation Trust, Herries Road, Sheffield S5 7AU, UK; 5 Department of Cardiovascular Sciences, University of Sheffield, Sheffield S10 2TN, UK; 6 Academic Urology Unit, K Floor, Department of Oncology, Royal Hallamshire Hospital, Glossop Road, University of Sheffield, Sheffield S11 7FE, UK.,

**Keywords:** prostate cancer, androgen deprivation, cost-effectiveness, cardiovascular risk

## Abstract

In the 70 years following the first description of the benefits of surgical castration, despite advances in medical therapy e.g. cabazitaxel, enzalutamide, abiraterone, androgen deprivation therapy (ADT) remains the cornerstone of treatment for advanced prostate cancer. However, with increasing numbers of men undergoing PSA testing, the disease is being diagnosed earlier and the costs of ADT, with uncertain survival benefits and associated risks, have risen dramatically. Clinical studies of potent novel agents have shown survival benefits in advanced disease, but timing, risks and cost-effectiveness of treatment remain controversial. As new agents enter clinical practice, a comprehensive research strategy is essential to optimise benefits whilst minimising harm.

## Introduction

**Figure 1:**
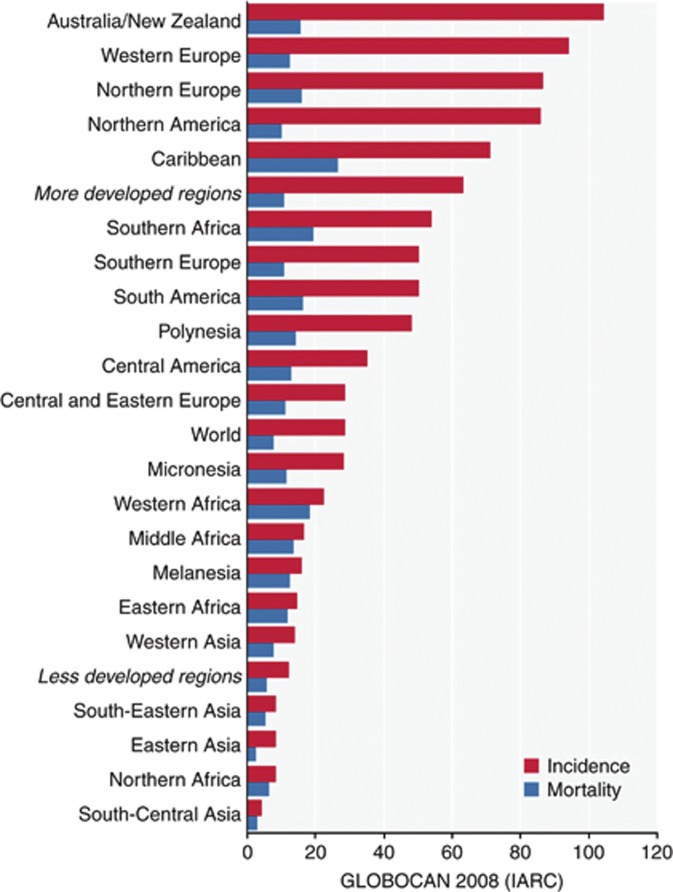
Estimated age-standardised rates of prostate cancer incidence and mortality per 1 00 000 men. Reproduced from globocan.iarc.fr April 2012.

Prostate cancer is the sixth most common cause of cancer mortality worldwide (268 000 deaths recorded in 2008). There is considerable discrepancy between reported incidence and mortality (Figure 1), particularly in the developed world attributable mainly to prostate specific antigen (PSA) testing. A significant number of men diagnosed with prostate cancer will survive for many years, even without radical curative treatment. Endocrine therapy is the mainstay of treatment for advanced prostate cancer. It has been reported that nearly 50% of all men diagnosed with prostate cancer will undergo androgen deprivation therapy (ADT) at some stage after diagnosis (Meng *et al*, 2002) and men remain on such treatment for up to two decades (Schroder *et al*, 2009). Despite trends in a reduction of inappropriate ADT use in the USA (Shahinian *et al*, 2010) the situation in other health care systems where these agents are widespread, such as the UK, remains uncertain. Part of the problem in evaluating the size of the issue is that in most countries, data on the precise timing of ADT in men with prostate cancer are not routinely collected. Guidelines produced by the European Association of Urology (Mottet *et al*, 2011), the National Comprehensive Cancer Network (NCCN, 2012), and The British Association of Urological Surgeons (BAUS, 2009) refer to caution being exercised with regards to timing of introduction of ADT in men with advanced prostate cancer, however controversy surrounds the concept of what constitutes ‘early’ or ‘late’ in this context.

Observational evidence has been accumulating suggesting that men on long-term conventional ADT may be at risk of treatment-related adverse events, in particular cardiovascular disease (CVD) (Bourke *et al*, 2011). Links have already been made between low testosterone levels (hypogonadism) and CVD in other settings (Malkin *et al*, 2010). The severe iatrogenic hypogonadal state induced by medical or surgical castration to treat prostate cancer could augment CVD risk in these men.

What of the costs of such therapy? With increasing lead-time bias and thus treatment duration, costs are spiralling upwards. Data from the 2010 NHS prescription cost analysis reports that around £100 million was spent on ADT in England (inclusive of Bicalutamide, Buserelin, Cyproterone Acetate, Degarelix, Flutamide, Goserelin Acetate, Leuprorelin Acetate and Triptorelin Acetate) (The NHS Information Centre PSU, 2010). This represents 28% of the total spend on malignant disease & immunosuppressive drugs. The situation in the United States is similar and this probably reflects the situation in every healthcare system in the developed world.

In the context of burgeoning costs and accumulating evidence of risk of ADT, closer scrutiny of the risks and benefits of ADT for advanced prostate cancer is warranted. This is particularly relevant given the recent introduction of the novel androgen synthesis inhibitor, abiraterone (de Bono *et al*, 2011) and reported similar improved survival results for enzalutamide, a first-in-class androgen inhibitor (Scher *et al*, 2012) in men with chemotherapy relapsed castrate-resistant prostate cancer. Demonstration of efficacy in such late-stage disease will certainly lead to investigation of these treatments earlier in the disease.

## The history of endocrine therapy in prostate cancer

Charles Huggins, (Huggins & Hodges, 1941) demonstrated the effectiveness of orchiectomy in palliating symptomatic metastatic prostate cancer. Several large studies carried out in the ‘60s and ‘70s (VACURG series) established the role of endocrine therapy in mainstream clinical practice by showing equivalent benefit of orchiectomy and of Diethylstilboestrol (DES) in providing effective palliation in end-stage disease, but evidence for improvement in survival remained elusive. With time, it became evident that high-dose DES was associated with excess cardiovascular mortality (de Voogt *et al*, 1986) which manifested as early as the first six months of treatment. First-line treatment with oestrogens was all but forgotten for the next 30 years.

The clinical picture in prostate cancer has been dramatically altered by prostate-specific antigen (PSA) testing and by the use of luteinizing hormone-releasing hormone (LHrH) analogues (Tolis *et al*, 1982; Waxman *et al*, 1983). On the one hand, men are being diagnosed earlier with prostate cancer creating significant lead-time bias. On the other, the availability of medical castration, apparently without excess morbidity (Waxman *et al*, 1983) has meant removal of the negative connotations of surgical castration, thus improving the acceptability of such treatment to both patients and clinicians. The result is that despite evidence of superior cost-effectiveness of surgical castration, LHrH agonists have become the predominant mode of castration, often instituted earlier in the disease than originally intended when castration for advanced prostate cancer was first described.

### Is ADT beneficial?

There is good evidence for the use of ADT, either in the form of orchiectomy or LHrH analogues, in advanced symptomatic metastatic prostate cancer. The benefits are mostly in palliation of symptoms. There have been very few studies comparing the use of endocrine therapy at any stage in prostate cancer to no hormonal therapy at all. As prostate cancer progresses and becomes symptomatic most men are placed on ADT. Hence, the existing research has sought to assess whether ‘early’ versus ‘late’ ADT prolongs life significantly. There is weak evidence of improved overall survival but only at 10 years with immediate ADT compared with deferred ADT (absolute risk reduction=5.5%) from a Cochrane review of early *vs*. deferred ADT in the treatment of advanced prostate cancer (Nair *et al*, 2002). However, the number of patients available for analysis at 10 years of follow-up was small and relied almost exclusively on a single VACURG study from the pre-PSA era using castration by orchiectomy rather than LHrH analogues (Jordan *et al*, 1977). Furthermore, the review authors reported important caveats that the trials which were synthesised had different control comparisons, heterogenous definitions of disease progression and none had validated assessments of prostate cancer specific mortality. As such, conclusive evidence of prostate cancer survival when ADT is initiated at diagnosis compared to waiting until emergence of symptomatic metastatic disease remains limited (Studer *et al*, 2006; Schroder *et al*, 2009). The updated results of EORTC 30891 comparing immediate ADT to deferred treatment initiated at the time of symptomatic disease progression or life-threatening complications showed a modest improvement in overall survival (HR= 1.21) favouring immediate treatment.(Studer *et al*, 2011) The cumulative mortality due to prostate cancer at 10-years was almost identical (22.2% and 21.0%) in the deferred and immediate arms respectively. It is important to point out that the study was originally designed to demonstrate non-inferiority of deferred treatment.

In contrast, the use of ADT in the context of multimodal therapy (i.e. in combination with radical prostatectomy or radiotherapy) has a more established evidence base (Kumar et al, 2006; Payne & Mason, 2011). ADT use of variable duration (from 6 months of ADT to orchietomy), in association with radical local treatment (e.g. for established lymphatic metastases following radical prostatectomy or in combination with radiotherapy in locally advanced disease), has been reported to improve overall and cancer specific survival. The evidence for radiotherapy is considerably stronger than that for surgery (Widmark *et al*, 2009; Verhagen *et al*, 2010). Two trials assessing combined radiotherapy with androgen deprivation have reported results of 71.5% overall survival at 5 years (Mottet *et al*, 2012) and a 39% cumulative overall mortality at 10 years (Widmark *et al*, 2009) in their ADT monotherapy arms. However, as neither trial was designed to assess incidence of iatrogenic harm or included a true control group i.e. a comparison of treatment without ADT, it is not possible to say whether ADT contributed significantly to the survival figures reported. There was a definite advantage in the group treated with combined ADT and radiotherapy over ADT alone at around 7 years of follow-up. Thus evidence for benefit in terms of efficacy can be said to exist when ADT is combined with radical local therapy, but only in men with sufficient life-expectancy to benefit. It is generally considered that the radio-sensitising effect of ADT is an important mechanism for improved outcomes in radiation therapy trials (Payne & Mason, 2011) rather than any effect on micrometastases.

### Can ADT do harm?

Outside the context of ADT in prostate cancer, there are multiple links between low androgen levels and CVD; low levels of androgens are commonly observed in patients with established coronary heart disease and heart failure. Over the last decade, various mechanisms whereby the effects of ADT could adversely affect CVD risk profile have been proposed including increase in body fat, reduction in lean mass, hyperlipidemia and changes in fasting plasma insulin/ fasting glucose levels.(Bourke *et al*, 2011) Observational evidence associates the use of ADT with an increased risk of diabetes, stroke, fatal and non-fatal myocardial infarction in men with prostate cancer. In an observational study of 14,597 men with local or regional prostate cancer (Keating *et al*, 2010) reported a significantly higher risk of diabetes (HR=1.28), coronary heart disease (HR=1.19), myocardial infarction (HR=1.28), sudden cardiac death (HR=1.35) and stroke (HR=1.22) on LHrH analogues when compared to men not taking these agents. Data from the Swedish cancer registry including 30,642 men with prostate cancer show a 40% higher rate of myocardial infarction in men undergoing primary ADT (Van Hemelrijck *et al*, 2010). A cohort study with nested case–control analysis from the UK General Practice Research Database (n=5103) also reported significantly elevated risks of coronary heart disease (OR=4.35), acute myocardial infarction (OR=3.57), heart failure (OR=3.19) and hospitalisation for heart failure (OR=3.39) in men undergoing combination therapy (LHrH agonists and anti-androgens) compared with men not on this treatment (Martin-Merino *et al*, 2011). This emerging evidence has led to a recent joint science advisory statement by the American Heart Association, The American Cancer Society and the American Urological Association asserting that it is possible that ADT could increase cardiovascular risk on the basis of its adverse impact on risk factors (Levine *et al*, 2010). The over-riding message is that a cause and effect relationship between ADT and increased risk of CVD remains a plausible hypothesis that is yet to be falsified.

Such evidence remains circumstantial and there are limitations to drawing conclusions from cancer registries as analysis of non-cancer outcomes can be problematic due to missing data and unmeasured confounders, for example the resolution of data on cardiovascular disease severity is often not recorded as assiduously as cancer-specific data. Given that prostate cancer is a disease of elderly men, with a protracted natural history and that CVD may take decades to manifest from a constellation of risk factors, these caveats are worth careful consideration when evaluating the observational data. Hence, it is still unclear whether this reflects a causal relationship.

### Is there direct evidence of a link between LHrH analogues and CVD morbidity/ mortality?

Whereas convincing observational data and plausible mechanisms have been reported, there is no level 1 evidence linking LHrH analogues with an increase in CVD mortality/ morbidity. However, cardiovascular safety was initially declared on the basis of use in 12 patients (Waxman *et al*, 1983) and no randomised controlled trial has ever assessed cardiovascular morbidity and/or mortality as a primary outcome in men undergoing ADT by LHrH analogues. A recent meta-analysis (Nguyen *et al*, 2011) sought to synthesise such evidence. The article, reported as a comparison of exposure to ADT with non-exposure in matched subjects, synthesised data from studies reporting CVD mortality as an adverse event rather than a primary outcome. The meta-analysis concluded that treatment with ADT is not associated with any increased risk of CVD mortality (HR=0.93, 95% CI=0.79,1.10), with the results being described as ‘reassuring’. However, as has been pointed out elsewhere (Blankfield, 2012) methodological flaws cast doubt over this conclusion. Almost half the men (1930/4141) came from RTOG 85-31, as reported by Efstathiou (Efstathiou *et al*, 2009) to investigate ‘lifelong’ exposure to ADT and from a multicentre European study (Studer *et al*, 2006) examining immediate versus deferred ADT. Previous publications from RTOG 85-31 have reported 72% (n=322) of men in the intervention arm as having discontinued ADT early (Souhami *et al*, 2009), thus leading to uncertainty as to duration of exposure. The comparator ‘control’ arm in the European study included men with substantial exposures to ADT, with around 25% of men in the deferred group being commenced on ADT within 3 years of randomisation.

We recalculated the meta-analysis summary statistic (D'Amico *et al*, 2008; Roach *et al*, 2008; Bolla *et al*, 2010; Denham *et al*, 2011) removing data from RTOG 85-31 (Efstathiou *et al*, 2009) and trials that compared immediate with deferred ADT (Messing *et al*, 2006; Studer *et al*, 2006; Schroder *et al*, 2009) (see Table 1), where there had been contamination of the control group. Importantly the median duration of ADT is only 6 months, which is considerably shorter exposure than is used in clinical practice for the majority of men on ADT. The result still indicates that for these combined radiotherapy trials, there is no significant increase in the risk of CVD mortality seen through the use of ADT (RR=1.06; 95% CI=0.80,1.40). The confidence interval is however wider than in the previous meta-analysis, as a result of having excluded the two largest trials and the conclusion is therefore open to question. The confidence interval of this, arguably more appropriate, pooled estimate is consistent with CVD risk being as much as 40% greater with ADT in some men. Given the observations above, can the scientific and clinical community and, above all, men with prostate cancer truly be reassured that CVD mortality is not a legitimate concern? And how should the risk of potential CVD morbidity be considered?

Based on the available data, it remains plausible that men on long-term ADT are at increased risk, but we do not reliably know the degree of risk, who is at risk nor how best to minimise this risk. The only research design that will give us definitive cause and effect evidence is a multicentre randomised controlled trial with primary cardiovascular morbidity/ mortality endpoints, however, given the circumstances, such a study is unlikely. In its absence, a bespoke synthesis of data from studies comparing men on long-term ADT to matched controls with high detail resolution on CVD outcomes to construct an individual patient data meta-analysis with integrated meta-regression to elucidate dose responses, could be highly informative. Without such studies, we are likely to be presented year after year with more observational data: all with the same inherent problems of bias due to, *inter alia*, non-randomisation, non-validation of CVD mortality, and incomplete assessment of pertinent co-morbidity.

## Is the treatment cost-effective?

Decisions made by NICE about whether cost effective treatments are approved for use in the NHS usually apply a threshold of around £20 000-£30 000 per QALY. Neither the Department of Health nor NICE hold any data on the cost-effectiveness or QALYs for conventional ADT drugs such as GNrH/LHrH analogues. Given the relative lack of evidence of benefit of long-term ADT, can we really justify spending nearly £100 m on a class of drugs, with an uncertain long-term safety profile?

It is inevitable, that should a man survive long enough on ADT, castrate-resistant prostate cancer will develop and until recently, few treatment options had been shown to be effective in improving overall survival in men with such disease. Taxane-based chemotherapy has been shown to be effective in this group of men, but is associated with significant toxicity. Several agents are currently showing promise in this group of men, with NICE having initially evaluated Abiraterone, an inhibitor of androgen synthesis at £63 200 per QALY and deeming it not to be cost-effective, further stating that the criteria for an end-of-life treatment were not met (for which the thresholds could be increased). This decision was reversed in June 2012 after the manufacturer (Janssen) has lowered the cost to an “undisclosed lower price”. Enzalutamide, a novel androgen receptor antagonist has recently been shown to be effective in prolonging survival in men with castrate-resistant prostate cancer following failure of taxane chemotherapy (Scher *et al*, 2012) Whereas such advances potentially represent good news for men with prostate cancer, it must be recognised, that the inclusion criteria into the studies has been on the basis of failure of docetaxel. Studies are now underway evaluating the role of Abiraterone in localised/ high risk disease (i.e. the addition of Abiraterone to the STAMPEDE trial). Prostate cancer, including castrate-resistant prostate cancer, represents a spectrum of disease from a rising PSA level through to rapidly progressive clinically-evident disease. Unless the indications (including precise stage of disease) for using these drugs are clearly defined in advance, as well as the potential toxicities associated with long-term use, significant migration in costs and possible adverse events, as has occurred with existing conventional ADT, may ensue. Therefore, a better understanding of the potential risks and any excess treatment cost associated with ADT is imperative to inform how cost effective these drugs truly are.

## Conclusions

There is good evidence that treatment of advanced prostate cancer by ADT results in improvements in symptoms in men with end-stage disease but, at best, weak evidence for improvement in survival, except when combined with radical local treatment, particularly radiotherapy. With the advent of PSA testing and more aggressive treatment, men are remaining on ADT for much longer than might have been originally anticipated. Newer, more potent agents have now become available, and although survival benefit for these has been shown in end-stage castrate-resistant disease, it is only a matter of time before they will be considered in earlier disease, possibly as a new form of maximal androgen blockade. Within this context, it seems reasonable to re-evaluate the evidence and consider questions for further studies to address. These include (i) Which stages of prostate cancer warrant treatment and with which agent? (ii) In which men with prostate cancer can any form of ADT be safely deferred? (iii) Do existing co-morbidities put men at increased CVD risk with ADT? (iv) Will more effective androgen deprivation, as possible with newer agents, result in higher risks when used for longer than has currently been investigated and how should these be measured? (v) What interventions mitigate any increased risk of CVD? (vi) What are the true benefits of treatment when the patient is viewed holistically and what are the full costs to the NHS, including treatment of adverse events associated with these drugs. The expansion of indications in the pursuit of cancer-specific benefit may result in men being on combined, highly potent, expensive androgen deprivation for many years, at great cost and with possible attendant risks. Therefore a better understanding of these risks and any excess treatment cost associated with ADT is imperative to inform how cost effective these drugs truly are and whether interventions are available to reduce their risks.

## Figures and Tables

**Table 1:** Re-calculation of a meta-analysis of CVD mortality due to use of AST first presented by Nguyen *et al* ^18^

		**AST**		**Control**			**Risk ratio**
**Study**	**Median AST duration (months)**	**Events**	**Total**	**Events**	**Total**	**Weight (%)**	**M-H, Random, 95%CI**
D’Amico *et al*, 2008	6	13	102	13	104	15.1	1.02 (0.50, 2.09)
Bolla *et al*, 2010	36	22	207	17	208	21.4	1.30 (0.71, 2.38)
Roach *et al*, 2008	4	31	224	26	232	32.7	1.23 (0.76, 2.01)
Denham *et al*, 2011	3–6	36	532	23	270	30.8	0.79 (0.48, 1.31)
Total (95% CI)			1065		814		1.06 (0.80,1.40)
Total events		102		79			

Heterogeneity: I^2^=0% (*P*=0.55)

Test for overall effect: Z=0.40 (*P*=0.69)
